# Investigation of metabolites for estimating blood deposition time

**DOI:** 10.1007/s00414-017-1638-y

**Published:** 2017-08-05

**Authors:** Karolina Lech, Fan Liu, Sarah K. Davies, Katrin Ackermann, Joo Ern Ang, Benita Middleton, Victoria L. Revell, Florence J. Raynaud, Igor Hoveijn, Roelof A. Hut, Debra J. Skene, Manfred Kayser

**Affiliations:** 1 Department of Genetic Identification, Erasmus MC University Medical Center Rotterdam, Rotterdam, The Netherlands; 2 Key Laboratory of Genomic and Precision Medicine, Beijing Institute of Genomics, Chinese Academy of Sciences, Beijing, China; 3 University of Chinese Academy of Sciences, Beijing, China; 4 Chronobiology, Faculty of Health and Medical Sciences, University of Surrey, Guildford, UK; 5 EaStCHEM School of Chemistry, Biomedical Sciences Research Complex and Centre of Magnetic Resonance, University of St. Andrews, St. Andrews, UK; 6 Cancer Research UK Cancer Therapeutics Unit, Division of Cancer Therapeutics, The Institute of Cancer Research, London, UK; 7 Groningen Institute for Evolutionary Life Sciences, Chronobiology unit, University of Groningen, Groningen, The Netherlands

**Keywords:** Blood deposition time, Metabolites, Circadian biomarkers, mRNA, Trace time estimation

## Abstract

Trace deposition timing reflects a novel concept in forensic molecular biology involving the use of rhythmic biomarkers for estimating the time within a 24-h day/night cycle a human biological sample was left at the crime scene, which in principle allows verifying a sample donor’s alibi. Previously, we introduced two circadian hormones for trace deposition timing and recently demonstrated that messenger RNA (mRNA) biomarkers significantly improve time prediction accuracy. Here, we investigate the suitability of metabolites measured using a targeted metabolomics approach, for trace deposition timing. Analysis of 171 plasma metabolites collected around the clock at 2-h intervals for 36 h from 12 male participants under controlled laboratory conditions identified 56 metabolites showing statistically significant oscillations, with peak times falling into three day/night time categories: morning/noon, afternoon/evening and night/early morning. Time prediction modelling identified 10 independently contributing metabolite biomarkers, which together achieved prediction accuracies expressed as AUC of 0.81, 0.86 and 0.90 for these three time categories respectively. Combining metabolites with previously established hormone and mRNA biomarkers in time prediction modelling resulted in an improved prediction accuracy reaching AUCs of 0.85, 0.89 and 0.96 respectively. The additional impact of metabolite biomarkers, however, was rather minor as the previously established model with melatonin, cortisol and three mRNA biomarkers achieved AUC values of 0.88, 0.88 and 0.95 for the same three time categories respectively. Nevertheless, the selected metabolites could become practically useful in scenarios where RNA marker information is unavailable such as due to RNA degradation. This is the first metabolomics study investigating circulating metabolites for trace deposition timing, and more work is needed to fully establish their usefulness for this forensic purpose.

## Introduction

Knowing the time of the day or night when a biological trace was placed at a crime scene has valuable implications for criminal investigation. It would allow verifying the alibi and/or testimony of the suspect(s) and could indicate whether other, yet unknown suspects may be involved in the crime. As such, knowing the trace deposition time would provide a link, or lack of, between the sample donor, identified via forensic DNA profiling, and the criminal event. Therefore, finding a means to retrieve information about the deposition time of biological material is of inestimable forensic value. In principle, molecular biomarkers with rhythmic changes in their concentration during the 24-h day/night cycle and analysible in crime scene traces would provide a useful resource for trace deposition timing.

Circadian rhythms are oscillations with a (near) 24-h period present in almost every physiological and behavioural aspect of human biology. They are generated on a molecular level by coordinated expression, translation and interaction of core clock genes and their respective protein products [1]. Together, these genes form a transcriptional-translational feedback loop driving the expression of various clock-controlled genes, which manifests as rhythms in numerous processes including metabolism [2–7], where circadian timing plays a role in coordinating biochemical reactions and metabolic activities. Because of this ubiquity of circadian rhythms and their association with many biological processes, the pool of potential rhythmic biomarkers is vast and diverse [8].

In a proof-of-principle study, we previously introduced the concept of molecular trace deposition timing, i.e. to establish the day/night time when (not since) a biological sample was placed at the crime scene, by measuring two circadian hormones, melatonin and cortisol, in small amounts of blood and saliva, and demonstrated that the established rhythmic concentration pattern of both biomarkers can be observed in such forensic-type samples [9]. Recently, we identified various rhythmically expressed genes in the blood [10] and subsequently demonstrated the suitability of such messenger RNA (mRNA) biomarkers for blood trace deposition timing by establishing a statistical model based on melatonin, cortisol and three mRNA biomarkers for predicting three day/night time categories: morning/noon, afternoon/evening and night/early morning [11].

Here, we investigate different types of molecular biomarkers, namely metabolites, i.e. intermediates or products of metabolism, for their suitability in trace deposition timing. Metabolic processes are known to be coupled with the circadian timing system in order to properly coordinate and execute them [6, 12, 13]. Thus, many (by-)products of metabolism have been shown to exhibit rhythms in their daily concentration levels in metabolomics studies [7, 14, 15], while none of them as yet have been tested for trace deposition timing. Using plasma obtained from blood samples collected every 2 h across a 36-h period from healthy, young males, 171 metabolites were screened via a targeted metabolomics approach to identifiy those with statistically significant rhythms in concentration. Rhythmic markers, as shown previously with hormones and mRNA [9, 11], are able to predict day/night time categories. Thus, we hypothesized that applying rhythmic metabolites (with or without previously established rhythmic biomarkers) for time prediction modelling could improve the categorical time prediction for trace deposition timing, which was assessed in this study.

## Materials and methods

### Metabolite data

The plasma metabolite data used in this study were obtained from blood samples collected during the sleep/sleep deprivation study (S/SD) conducted at Surrey Clinical Research Centre (CRC) at the University of Surrey, UK. Full details of the study protocol and eligibility criteria have been reported elsewhere [4, 5, 7]. For the present analysis, 18 sequential two-hourly blood samples per participant (*n* = 12 males, mean age ± standard deviation = 23 ± 5 years) were used, giving a total of 216 observations for subsequent model building. These samples spanned the first 36 h of the S/SD study (from 12:00-h day 2 to 22:00-h day 3). The samples covering the subsequent sleep deprivation condition, from 00:00 h on day 3 to 12:00 h on day 4, were excluded from the analysis. Full details of the blood sample collection, plasma extraction method, targeted LC/MS metabolomics analysis and subsequent statistical analyses have been described in Materials and Methods and Supplementary Material sections of the previous articles [4, 5, 7]. Concentration data of 171 metabolites (μM), belonging to either acylcarnitines, amino acids, biogenic amines, hexose, glycerophospholipids and sphingolipids, were obtained using the Absolute*IDQ* p180 targeted metabolomics kit (Biocrates Life Sciences AG, Innsbruck, Austria) run on a Waters Xevo TQ-S mass spectrometer coupled to an Acquity HPLC system (Waters Corporation, Milford, MA, USA).

After correcting the metabolite data for batch effect described in detail in [7], we analysed the metabolite profiles with the single cosinor and nonlinear curve fitting (nlcf) methods to determine the presence of 24 h rhythmicity, as was done previously [4, 5]. This first selection step of metabolites for time category prediction was based on the statistically significant outcomes from the nlcf and single cosinor methods. The selected metabolites had to have a statistically significant amplitude and acrophase, calculated with the nlcf method, and statistically significant fits to a cosine curve, as calculated with the single cosinor method.

### Model building and validation

Final selection of markers for prediction modelling was done using multiple regression including all markers as the explainary variables and the sampling time as the dependent variable and ensuring all of the selected markers having statistically significant and independent effect on the overall model fitting. The metabolite markers that did not show a statistically significant independent effect were excluded from the marker selection process. The most suitable predicted time categories were established, based on the average peak times of the metabolites and hormone concentrations, as calculated with the nlcf method.

The prediction model was built based on multinomial logistic regression, where the batch-corrected concentration values of metabolites were considered as the predictors and the day/night time categories as the response variable, as described elsewhere [11, 16]. Additionally, we combined the previously proposed circadian hormones melatonin and cortisol [9] as well as the previously established rhythmic mRNA biomarkers *MKNK2*, *PER3* and *HSPA1B* [11] with the metabolites in a prediction model, to determine whether a combination of the different types of rhythmic markers improves the prediction accuracy of time estimations. The dataset used for prediction modelling consisted of 216 observations, i.e. 12 individuals and 18 time points per individual. The multinomial logistic regression is written as

$$ \mathrm{logit}\left(\mathit{\Pr}\left(y= morningnoon\ |{x}_1\dots {x}_k\right)\right)=\mathit{\ln}\left(\frac{\pi_1}{\pi_3}\right)={\alpha}_1+\sum \beta {\left({\pi}_1\right)}_k{x}_k $$

$$ \mathrm{logit}\left(\mathit{\Pr}\left(y= afternooneve\ |{x}_1\dots {x}_k\right)\right)=\mathit{\ln}\left(\frac{\pi_2}{\pi_3}\right)={\alpha}_2+\sum \beta {\left({\pi}_2\right)}_k{x}_k $$

and the probabilities for a certain day/night category can be estimated as

$$ {\pi}_1=\frac{\mathit{\exp}\left({\alpha}_1+\sum \beta {\left({\pi}_1\right)}_k{x}_k\right)}{1+\mathit{\exp}\Big({\alpha}_1+\sum \beta \left({\pi}_1\Big){}_k{x}_k\right)+\mathit{\exp}\left({\alpha}_2+\sum \beta {\left({\pi}_2\right)}_k{x}_k\right)} $$

$$ {\pi}_2=\frac{\mathit{\exp}\left({\alpha}_2+\sum \beta {\left({\pi}_2\right)}_k{x}_k\right)}{1+\mathit{\exp}\Big({\alpha}_1+\sum \beta \left({\pi}_1\Big){}_k{x}_k\right)+\mathit{\exp}\left({\alpha}_2+\sum \beta {\left({\pi}_2\right)}_k{x}_k\right)} $$

and *π*_3_ = 1 − *π*_1_ − *π*_2_.

The day and night category with the max (*π*_1_ , *π*_2_ , *π*_3_) was considered as the predicted time category.

The model predicted the probabilities of different possible outcomes of a categorical dependent variable, given a set of variables (predictors), as previously described and applied for eye and hair colour prediction based on SNP genotypes [16–18] and for trace deposition time using circadian mRNA biomarkers [11].

Because of the small sample size, the performance of the generated model(s) was evaluated using the leaving-one-out cross-validation (LOOCV) method [19]. This approach builds a prediction model from all observations minus one, in this case for 215 observations, and predicts the time category for the one remaining observation. The whole procedure is repeated once for each observation, i.e. in this case 216 times. The area under the receiver operating characteristic (ROC) curve (AUC), which describes the accuracy of the prediction, was derived for each time category based on the concordance between the predicted probabilities and the observed time category. In general, AUC values range from 0.5, which corresponds to random prediction, to 1.0, which represents perfect prediction. The concordance between the predicted and observed categories was categorized into four groups: true positives (TP), true negatives (TN), false positives (FP) and false negatives (FN). Four accuracy parameters were derived: sensitivity = TP / (TP + FN) × 100, specificity = TN / (TN + FP) × 100, positive predictive value (PPV) = TP / (TP + FP) × 100 and negative predictive value (NPV) = TN / (TN + FN) × 100.

Notably, the 216 observations that were used in this study were not completely independent from each other; however, we aimed to minimize the bias by cross-validation using LOOCV.

## Results

### Identification of rhythmic metabolites and biomarker selection for time prediction modelling

From the 171 metabolites analysed in the plasma samples, we identified 56 metabolite biomarkers showing statistically significant oscillations, with both the nlcf and cosinor methods (Table 1). Next, these 56 metabolites were assigned to day or night time categories based on their mean peak (acrophase) time estimates (Table 1). An overrepresentation of metabolites (*n* = 50, 89%) demonstrating peak concentrations in the afternoon, between 13:00 and 17:30 h, was noted. Five out of 56 (9%) metabolites had their highest concentration values during the night, between 21:00 and 03:00 h. Only one metabolite showed a peak time in the early morning, around 06:00 h. Consequently, we assigned all 56 metabolites to three day/night time categories, i.e. morning/noon (07:00–14:59 h), afternoon/evening (15:00–22:59 h) and night/early morning (23:00–06:59 h), together comprising one complete 24-h day/night cycle.

**Table 1:** Plasma metabolites (*n* = 56) with statistically significant rhythmicity in concentration during the 24-h day/night cycle, identified using both the single cosinor and nonlinear curve fitting (nlcf) methods, with their assigned time categories

Number	Metabolite	Average acrophase (single cosinor) (time in h)	Average acrophase (NLCF) (time in h)	Assigned time category
1	AC-C0	6.45	6.16	Morning/noon
2	AC-C14	16.18	16.52	Afternoon/evening
3	AC-C14:2	14.01	13.40	Afternoon/evening
4	AC-C16	15.14	15.31	Afternoon/evening
5	AC-C16-OH	16.11	16.43	Afternoon/evening
6	AC-C16:1-OH	13.40	15.49	Afternoon/evening
7	AC-C16:2-OH	13.39	14.49	Afternoon/evening
8	AC-C18:1	13.12	13.08	Afternoon/evening
9	AC-C18:2	13.49	13.41	Afternoon/evening
10	lysoPC a C16:0	15.04	15.31	Afternoon/evening
11	lysoPC a C18:0	14.56	15.34	Afternoon/evening
12	PC aa C36:4	16.29	17.13	Afternoon/evening
13	PC aa C38:0	14.09	14.25	Afternoon/evening
14	PC aa C38:3	14.39	15.20	Afternoon/evening
15	PC aa C38:4	15.21	15.52	Afternoon/evening
16	PC aa C38:5	14.56	15.38	Afternoon/evening
17	PC aa C38:6	15.01	15.40	Afternoon/evening
218	PC aa C40:1	13.28	14.27	Afternoon/evening
19	PC aa C40:2	13.50	14.44	Afternoon/evening
20	PC aa C40:3	14.13	15.00	Afternoon/evening
21	PC aa C40:4	14.34	14.57	Afternoon/evening
22	PC aa C40:5	14.42	15.07	Afternoon/evening
23	PC aa C40:6	14.43	15.23	Afternoon/evening
24	PC aa C42:0	13.57	14.17	Afternoon/evening
25	PC aa C42:1	14.04	14.43	Afternoon/evening
26	PC aa C42:2	13.49	14.45	Afternoon/evening
27	PC aa C42:4	14.19	14.48	Afternoon/evening
28	PC aa C42:5	14.20	14.54	Afternoon/evening
29	PC aa C42:6	14.12	14.47	Afternoon/evening
30	PC ae C32:2	13.20	14.20	Afternoon/evening
31	PC ae C34:2	14.25	15.51	Afternoon/evening
32	PC ae C36:3	14.16	15.05	Afternoon/evening
33	PC ae C36:4	14.19	14.25	Afternoon/evening
34	PC ae C36:5	14.22	14.10	Afternoon/evening
35	PC ae C38:0	14.58	16.15	Afternoon/evening
36	PC ae C38:4	13.42	14.10	Afternoon/evening
37	PC ae C38:5	14.13	14.25	Afternoon/evening
38	PC ae C38:6	14.08	14.23	Afternoon/evening
39	PC ae C40:1	14.33	16.43	Afternoon/evening
40	PC ae C40:3	13.22	14.04	Afternoon/evening
41	PC ae C40:4	13.57	14.30	Afternoon/evening
42	PC ae C40:5	13.54	14.19	Afternoon/evening
43	PC ae C40:6	13.28	14.03	Afternoon/evening
44	PC ae C42:0	14.29	15.14	Afternoon/evening
45	PC ae C42:4	14.16	14.49	Afternoon/evening
46	PC ae C42:5	14.06	14.25	Afternoon/evening
47	PC ae C44:4	14.06	14.37	Afternoon/evening
48	PC ae C44:5	14.17	14.29	Afternoon/evening
49	PC ae C44:6	14.16	14.23	Afternoon/evening
50	SMC16:1	13.28	13.39	Afternoon/evening
51	SMC24:1	13.51	13.51	Afternoon/evening
51	AC-C4	2.48	2.37	Night/early morning
53	Isoleucine	22.45	22.36	Night/early morning
54	Proline	21.16	21.07	Night/early morning
55	Sarcosine	21.58	22.03	Night/early morning
56	lysoPC a C18:2	20.48	21.12	Night/early morning

Metabolite order based on the assigned time category

*AC* acylcarnitines, *lysoPC a* lysophosphatidylcholines, *PC aa* diacylphosphatidylcholines, *PC ae* acyl-alkyl-phosphatidylcholines, *SM* sphingomyelins

### Time prediction modelling using metabolites and other biomarkers

**Fig. 1:**
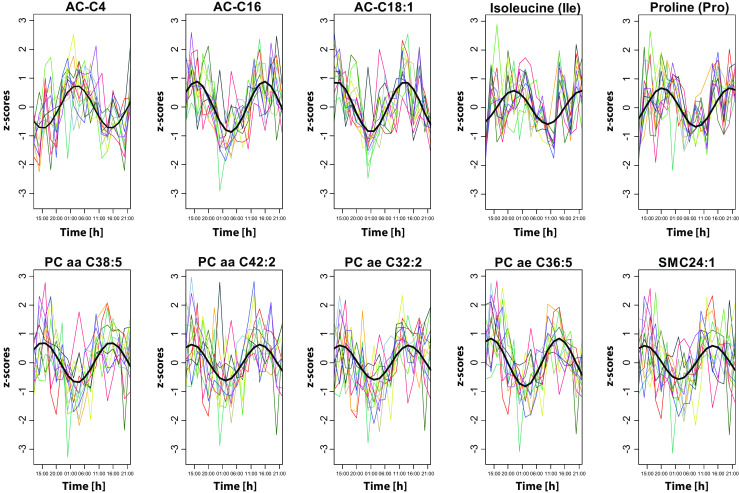
Ten rhythmic metabolite markers selected for time prediction modelling. The data are presented as *z* scores (for illustrative purposes only) across a period of 36 h. Each *coloured line* represents one individual; the *black bold line* corresponds to an average cosine curve (as calculated with the nlcf method)

In the first step of the biomarker selection, we applied linear regression to all 56 metabolites, identified as significantly rhythmic, to select those with an independent contribution to the model for predicting the three day/night time categories: morning/noon, afternoon/evening and night/early morning, as previously done for mRNA and hormone biomarkers [11]. This analysis revealed a subset of 10 metabolite biomarkers (AC-C16, AC-C18:1, AC-C4, isoleucine, proline, PCaaC38:5, PCaaC42:2, PCaeC32:2, PCaeC36:5 and SMC24:1). The remaining 46 metabolites were omitted from the subsequent model building and model validation analysis as their effect on time category prediction was ‘masked’ by the 10 metabolite biomarkers included in the model. A multinomial logistic regression model including the 10 independently contributing metabolite biomarkers achieved cross-validated AUC values of 0.81, 0.86 and 0.90 for the three time categories: morning/noon, afternoon/evening and night/early morning respectively (for the outcomes of other prediction accuracy parameters, see Table 2). Figure 1 presents *z*-scored concentration values across the day/night cycle for these 10 metabolite biomarkers.

**Table 2:** Accuracy estimates of time prediction models based on significantly rhythmic and independently contributing biomarkers

Model based on metabolites AC-C16, AC-C18:1, AC-C4, isoleucine, proline, PC aa C38:5, PC aa C42:2, PC ae C32:2, PC ae C36:5, SMC24:1					
Predicted time category	AUC	Sens	Spec	PPV	NPV
Morning/noon	0.81	0.55	0.85	0.65	0.79
Afternoon/evening	0.86	0.82	0.77	0.75	0.84
Night/early morning	0.90	0.67	0.90	0.65	0.90
Model based on metabolites, hormones^a^ and mRNAs^a^ AC-C16, AC-C18:1, AC-C4, isoleucine, SMC24:1, melatonin^a^ and MKNK2^a^					
Predicted time category	AUC	Sens	Spec	PPV	NPV
Morning/noon	0.85	0.71	0.84	0.69	0.85
Afternoon/evening	0.89	0.78	0.82	0.78	0.83
Night/early morning	0.96	0.70	0.93	0.76	0.91

*AUC* area under the receiver operating characteristic (ROC) curve, *PPV* positive predictive value, *NPV* negative predictive value, *Spec* specificity, *Sens* sensitivity

^a^As established previously [11]

However, our previously established model based on two circadian hormones (melatonin and cortisol) and three mRNA biomarkers (*MKNK2*, *HSPA1B* and *PER3*) gave considerably higher AUC values of 0.88, 0.88 and 0.95 for the same three time categories respectively [11], than achieved here with the model based on the 10 plasma metabolites. Therefore, we performed time prediction modelling using the 10 metabolite biomarkers highlighted here together with the previously identified hormone and mRNA biomarkers. This analysis revealed a subset of seven independently contributing biomarkers: five metabolites (AC-C16, AC-C18:1, AC-C4, isoleucine and SMC24:1), one hormone (melatonin) and one mRNA biomarker (*MKNK2*). The AUC values obtained with this combined biomarker model were 0.85 for morning/noon, 0.89 for the afternoon/evening and 0.96 for night/early morning (Table 2).

## Discussion

In this forensically motivated metabolomics study, 56 metabolite biomarkers exhibiting significant daily rhythms in concentration were identified in plasma and were further investigated for their suitability for estimating blood trace deposition time. The 171 metabolites initially tested were included in the Absolute*IDQ* p180 targeted metabolomics kit (Biocrates Life Sciences AG, Innsbruck, Austria) and belong to five compound classes and are involved in major metabolic pathways, such as energy metabolism, ketosis, metabolism of amino acids, cell cycle and cell proliferation and carbohydrate metabolism, to name a few. Metabolism is interconnected with circadian rhythms, influencing them and, in turn, being influenced by them [2, 6, 12, 13, 20]. Among the metabolites with statistically significant oscillations identified here, we found a strong overrepresentation of those exhibiting peak concentrations in the afternoon, mainly from the phosphatidylcholine class (Table 1). Although currently we cannot fully understand what causes this overrepresentation, the observed peak times agree with data showing lipid metabolism transcripts in humans having maximum transcription levels during the day [21].

The prediction model established here utilized 10 metabolite biomarkers for estimating three day/night time categories and achieved AUCs of 0.81 for morning/noon, 0.86 for afternoon/evening and 0.90 for night/early morning categories. Following this finding, the metabolite model was compared to the models we previously introduced: (i) the hormone-based model, (ii) the mRNA-based model and (iii) the combined hormone and mRNA-based model [11]. The first comparison with the hormone-based model (cortisol and melatonin) (i) showed an improved performance of the metabolite-based model in predicting afternoon/evening and night/early morning categories (0.86 vs 0.83 and 0.90 vs 0.85 respectively) [11]. Comparison with mRNA-based model (biomarkers *HSPA1B*, *PER1*, *PER3*, *TRIB1*, *THRA1*, *MKNK2*) (ii) revealed that the metabolite-based model achieved higher AUCs for the morning/noon and afternoon/evening categories (0.81 vs 0.75 and 0.86 vs 0.80 respectively) [11]. In both model comparisons (i and ii), the remaining category was predicted slightly less accurately in the metabolite-based model. However, the final comparison with the combined model, based on two hormones (melatonin, cortisol) and three mRNA biomarkers (*MKNK2*, *HSPA1B* and *PER3*), (iii) showed that the metabolite-based model was considerably less accurate, giving lower AUC values by 0.07, 0.02 and 0.05, for morning/noon, afternoon/evening and night/early morning respectively [11]. This final finding was the motivation to combine together in one time prediction model the 10 metabolite biomarkers identified here, with the hormone and mRNA biomarkers identified previously [11]. The best combined model was based on five metabolites (AC-C16, AC-C18:1, AC-C4, isoleucine and SMC24:1), melatonin and the *MKNK2* and reached AUC values of 0.85 for morning/noon, 0.89 for afternoon/evening and 0.96 for night/early morning. Overall, this combined model was slightly more accurate in predicting the afternoon/evening and the night/early morning categories (AUC increase of 0.01) and slightly less accurate in predicting the morning/noon category (AUC decrease of 0.03) compared with the previously established combined hormone and mRNA-based model [11]. This rather minor impact of the newly tested metabolites, relative to the previously tested hormones and mRNA biomarkers [11], questions the value of using plasma metabolites for trace deposition timing.

The major subset of the metabolites identified in the current study peaked during the day, and this might reflect either the feeding-fasting schedule [7, 22] or their original source. The original source of metabolites circulating in plasma is difficult to determine accurately since they can be derived from multiple organs that are regulated by different systemic and external cues influencing their function and rhythmicity, which, in turn, modifies the rhythms of the generated metabolites. Consequently, if the metabolites identified here are sensitive to feeding and fasting cues, their applicability for trace deposition timing may be rather limited, but their value for monitoring peripheral circadian rhythms in the liver, for instance, may be crucial.

Furthermore, the previously introduced hormone and mRNA biomarkers [11] can feasibly be analysed by using an ELISA assay and RT-qPCR respectively, techniques that nowadays are straightforward and require only basic laboratory instruments and have been shown to be suitable for forensic trace analysis. In comparison, relatively specialized LC/MS equipment and methodology are needed to simultaneously analyse a large number of metabolites circulating in plasma, even more so, when measuring a forensic trace sample. Regardless of these constraints, it has been shown that measuring metabolites in dried blood is possible [23, 24], but needs to be studied further in the forensic context, where the quantity and the quality of dried blood stains are often compromised. However, in situations where intact RNA is not available and the preferred mRNA-based time estimation models can therefore not be used, metabolite markers might be the markers of choice. In such situation, metabolite analysis may provide valuable information on trace deposition time.

The technical challenges should thus not impede future studies to fully establish whether plasma metabolites could be useful biomarkers for trace deposition timing, and if additional metabolites can achieve a more detailed and accurate time estimation than the metabolites identified here. Additionally, more samples collected around the 24-h clock from more individuals need to be analysed to make the time prediction model more robust, and the analysis method, at best a multiplex system, needs to be forensically validated including sensitibity testing, specificity testing and stability testing, before final forensic casework application may be considered.
